# Reactivation of a loss-of-function *SlGLK2* allele by frame-restoring genome editing in tomato

**DOI:** 10.1007/s00299-026-04024-z

**Published:** 2026-10-15

**Authors:** Kentaro Ezura, Cheolwoo Park

**Affiliations:** Biological Resources and Post-Harvest Division, Japan International Research Center for Agricultural Sciences (JIRCAS), Tsukuba, Ibaraki 305-8686 Japan

**Keywords:** *GOLDEN2-LIKE*, Fruit development, Plastid, Tomato (*Solanum lycopersicum*), Genome editing

## Abstract

**Key message:**

Restoration of the crop improvement-related loss-of-function gene, *Solanum lycopersicum GOLDEN2-LIKE*, through an additional mutation by a conventional genome editing technique.

**Abstract:**

Plant domestication and crop improvement processes have inadvertently led to the loss of gene functions that contribute to crop quality. In widely cultivated tomato (*Solanum lycopersicum*) varieties, the *uniform ripening* (*u*) mutation, a loss-of-function allele of the *Solanum lycopersicum Golden2-like 2* (*SlGLK2*), has been selected to improve fruit appearance. However, this selection is associated with reduced fruit nutritional quality, lowering the levels of sugars and tocopherols due to impaired plastid development. In this study, the function of *SlGLK2* was restored by introducing an additional mutation using the temperature-tolerant *Lachnospiraceae bacterium* ND 2006 Cas12a (*Lb*Cas12a) genome editing system. A 13-bp deletion in the linker region of SlGLK2 protein in the *CR-Slglk2-4* line restored the reading frame and led to enhanced chlorophyll accumulation and plastid development, particularly in the stem end region of the fruit. The edited line also showed increased total soluble solids and tocopherol content in ripe fruit. Biochemical, transcriptomic, and transcriptional activity analyses confirmed the functional restoration of *SlGLK2* in the *CR-Slglk2-4* line. This study not only provides a useful Micro-Tom line for investigating plastid biogenesis in tomato fruit but also demonstrates the potential of genome editing to restore the latent functions of pseudogenized genes in modern crops, offering a new approach for recovering traits lost during plant domestication or crop improvement.

**Supplementary Information:**

The online version contains supplementary material available at https://doi.org/10.1007/s00299-026-04024-z.

## Introduction

Plant domestication and crop improvement have focused on selecting traits that are directly noticeable to humans or essential for cultivation under specific conditions. This process has often resulted in the fixation of alleles that enhance the agronomic performance at the cost of losing other alleles associated with traits that were not selected for, a phenomenon recognized as domestication-associated genetic trade-offs (Singh and van der Knaap 2022; Glaus et al. 2025). For instance, traits such as disease resistance (Gao et al. 2019), salt stress tolerance (Wang et al. 2020), drought tolerance (Li et al. 2024), root architecture (Singh et al. 2019), and nutritional value of fruit (Powell et al. 2012; Nguyen et al. 2014; Lupi et al. 2019) were overlooked during initial plant domestication or crop improvement efforts. Although introgression from wild relatives has been used to improve lost traits (Tanksley and McCouch 1997), this strategy often introduces undesirable linked loci, making it a complex and occasionally inefficient process.

Plastid development in tomato fruits is a notable example of crop improvement-associated trade-offs. Plastids contribute not only to photosynthesis but also to the biosynthesis of various nutritional metabolites such as carotenoids, sugars, and vitamins (Li et al. 2022; Mesa and Munné-Bosch 2023). The *GOLDEN2-LIKE* (GLK) transcription factors, which belong to the GARP family of MYB transcription factors, play a key role in regulating plastid development (Fitter et al. 2002; Waters et al. 2009; Zubo et al. 2018; Hernández‐Verdeja and Lundgren 2024). In tomato, *SlGLK1* and *SlGLK2* transcription factors govern chloroplast development in the leaves and fruits, respectively (Powell et al. 2012). The recessive *uniform ripening* (*u*) allele, a single nucleotide insertion in the coding region of the *SlGLK2* gene*,* leads to a premature stop codon and loss of function, resulting in a reduction in the number of plastids in the fruit (Powell et al. 2012; Nguyen et al. 2014). Although this mutation has been widely adopted in breeding programs to eliminate the green shoulder phenotype and improve visual uniformity, it compromises the accumulation of key nutritional compounds (Powell et al. 2012; Nguyen et al. 2014; Lupi et al. 2019).

Genome editing using site-directed nucleases (SDNs), particularly the clustered regularly interspaced short palindromic repeat (CRISPR)/Cas9 system, represents a powerful tool for the precise modification of target genes (Wang and Doudna 2023). SDN-based genome editing is categorized into three types: SDN1 to SDN3 (Podevin et al. 2013). Most practical applications to date have used the SDN1 category, which involves the induction of single-point mutations or insertions/deletions (InDels) (Menz et al. 2020). Various studies have focused on enhancing agronomic traits by inducing loss-of-function mutations in specific target genes (Chen et al. 2019; Wang et al. 2019). In contrast, reports demonstrating gain-of-function mutations are limited. For instance, CRISPR/Cas9-mediated deletion of the autoinhibitory domain in the glutamate decarboxylase 3 (GAD3) protein enhanced its activity, thereby elevating γ-aminobutyric acid (GABA) level in tomato fruit (Nonaka et al. 2017). Similarly, CRISPR/Cas9-mediated editing of the *DELLA/PROCERA* gene resulted in a gibberellin-responsive, dominant dwarf allele (Zhu et al. 2018; Tomlinson et al. 2019). A recent study further demonstrated that base editing can repair a deleterious domestication variant, enabling early fruit yields through the modification of shoot and inflorescence architecture (Glaus et al. 2025). Genome editing can therefore be applied not only to disrupt functions but also to optimize gene activity. However, the targeted restoration of a loss-of-function allele has rarely been demonstrated.

The current study demonstrates that genome editing can be used not only to disrupt but also to restore the gene function lost during tomato improvement process. A compensatory frameshift introduced through a temperature-tolerant *Lb*Cas12a system reconstituted SlGLK2 activity and rescued plastid development in fruits. These findings highlight a conceptual strategy in which genome editing of the SDN1 category can be used to revive gene functions lost during plant domestication or crop improvement and provide a framework for recovering valuable horticultural traits.

## Materials and methods

### Plant materials and growth conditions

The *Solanum lycopersicum* model cultivar ‘Micro-Tom’ carrying the non-functional *u* allele was used in this study. Plants were grown in a constant-temperature cultivation chamber at 24 °C with a photoperiod of 16 h light/8 h dark.

### Vector construction of a genome editing vector

All primers used in this study are listed in Table S1. To construct the pDe-tt*Lb*Cas12a_NTPII vector, the pDe-CAS9_NTPII vector, which originated from pDe-CAS9 (Addgene plasmid # 61,433) (Fauser et al. 2014), was linearized by *AscI* digestion and purified using the FastGene Gel/PCR Extraction Kit (NIPPON Genetics, Tokyo, Japan). A fragment of temperature-tolerant *Lb*Cas12a (tt*Lb*Cas12a), harboring the single mutation D156R, was obtained from pDe-EC-tt*Lb*Cas12a (Schindele and Puchta 2020) after *Asc*I digestion. The pDe-Cas9, pDe-EC-tt*Lb*Cas12a, and pEn-RZ-*Lb*-Chimera vectors were kindly provided by Dr. Holger Puchta (Karlsruhe Institute of Technology, Germany) (Schindele and Puchta 2020). The pDe-Cas9_NTPII was generated by inserting a synthetic NTPII cassette into the *Hind*III-digested pDe-Cas9 fragment using NEBuilder HiFi DNA Assembly Master Mix (New England Biolabs, Ipswich, MA, USA). The tt*Lb*Cas12a fragment was purified by gel extraction using the FastGene Gel/PCR Extraction Kit (NIPPON Genetics, Tokyo, Japan) and inserted into the *Asc*I-digested pDe-CAS9_NTPII vector using Ligation Mix (Takara, Shiga, Japan). Two complementary oligos containing a 24-bp CRISPR RNA (crRNA)-target sequence were annealed and cloned into the *Bbs*I-digested pEn-RZ-*Lb*-Chimera vector using Ligation Mix (Takara, Shiga, Japan). The crRNA is under the control of the Arabidopsis U6-26 promoter and flanked by the hammerhead and hepatitis delta virus ribozyme at the 5′ and 3′ sites, respectively. The crRNA cassette was confirmed by restriction enzyme analysis and sequencing. Finally, the crRNA cassette was transferred to the pDe-tt*Lb*Cas12a_crRNA:SlGLK2_NTPII vector by the LR reaction using the Gateway LR Clonase II (Thermo Fisher Scientific, Waltham, MA, USA).

### *Agrobacterium*-mediated transformation and isolation of mutants

The recombinant vector pDe-tt*Lb*Cas12a_crRNA:SlGLK2_NTPII was introduced into *Agrobacterium tumefaciens* strain GV2260 and transformed into tomato cv. Micro-Tom according to a previously described procedure (Sun et al. 2006). In the T_0_ generation, T-DNA positive lines were first isolated by polymerase chain reaction (PCR) of the tt*Lb*Cas12a region in T-DNA using gene-specific primers. To confirm the presence of mutations in *SlGLK2*, the sequence was confirmed using PCR amplification of the target region and subsequent direct sequencing of the PCR products. Transgene-negative homozygous alleles were verified in the T_1_ generation using PCR-based sequencing.

Potential off-target sites of the ttLbCas12a crRNA were predicted using Cas-OFFinder-bulge, a wrapper script for Cas-OFFinder (Bae et al. 2014). The tomato SL4.0 reference genome sequence was used as the search database. Because ttLbCas12a is derived from LbCas12a, the canonical Cas12a PAM pattern TTTV was specified. Candidate sites were searched under two conditions: up to three mismatches without bulges, and up to three mismatches with up to a one-nucleotide DNA or RNA bulge. The genomic position of the detected candidate site was compared with ITAG4.0 gene models to determine whether it overlapped with annotated gene features or nearby genes.

### Sequence analysis

Conserved domains and disordered regions were searched using InterProScan analysis (Quevillon et al. 2005). Nuclear localization signal (NLS) prediction was conducted using LOCALIZER (Sperschneider et al. 2017) and NLStradamus (Nguyen Ba et al. 2009). AlphaFold3 was used to predict the structure of SlGLK2 protein (Abramson et al. 2024).

### Sub-cloning of *SlGLK2* gene and sequence verification of SlGLK2 coding sequence

Total RNA was extracted from immature green fruit of Micro-Tom (*u* allele) and *CR-Slglk2-4* line (13-bp deleted allele) using an RNeasy Plant Mini Kit (Qiagen, Venlo, The Netherlands). First-strand cDNA was synthesized using PrimeScript RT reagent (Takara, Shiga, Japan). The full-length coding sequences (CDSs) were amplified from the cDNA mix using KOD ONE (Toyobo, Tokyo, Japan) and gene-specific primers (Table S1). The amplified CDS fragments were cloned into a PCR-amplified pENTR vector using NEBuilder HiFi DNA Assembly Master Mix (New England Biolabs, Ipswich, MA, USA) to construct pENTR-SlGLK2-*u* and pENTR-SlGLK2-CR4. The functional SlGLK2-u CDS was generated from pENTR-SlGLK2-*u* by PCR-based site-directed mutagenesis using gene-specific primers, followed by assembly with the NEBuilder HiFi DNA Assembly Master Mix. The sequences of all three constructs were verified by Sanger sequencing.

### Subcellular localization and transactivation assays of SlGLK2

To investigate the subcellular localization of functional and mutant SlGLK2 proteins, superfolder green fluorescent protein (sfGFP)-tagged expression vectors were constructed using the pGreenII62-SK backbone. First, a synthetic P19 silencing suppressor sequence was cloned into the *Xba*I and *Kpn*I sites of pGreenII62-SK (Hellens et al. 2000) to generate pGII62SK-P19. The P19 expression cassette was subsequently PCR-amplified and inserted into the *BamH*I and *Hind*III sites of the helper plasmid pSoup (Hellens et al. 2000) using the NEBuilder HiFi DNA Assembly Master Mix, resulting in pSoup-P19_K. This construct was introduced into the *A. tumefaciens* strain GV3101 (MP90) by electroporation and used as the co-infiltration strain. The synthetic sfGFP gene was cloned into the pGreenII62-SK vector. The *SlGLK2-U*, SlGLK2-*u*, and SlGLK2-*CR4* were amplified from the sequence-verified pENTR constructs and inserted upstream of sfGFP in-frame, producing pGII62SK-SlGLK2-*U*:sfGFP, pGII62SK-SlGLK2-*u*:sfGFP, and pGII62SK-SlGLK2-*CR4*:sfGFP, respectively. These constructs were individually transformed into *Agrobacterium* GV3101, which carried pSoup-P19_K.

Agroinfiltration was conducted according to a standard protocol (Hellens et al. 2000) as follows. The transformed *Agrobacterium* strains were grown overnight, and 1 mL of the cultures was inoculated into 20 mL of Luria–Bertani (LB) broth supplemented with 50 mg/L kanamycin, 50 mg/L rifampicin, 10 mg/L gentamicin, and 15 µM acetosyringone. The culture was incubated at 28 °C until optical density at 600 nm (OD_600_) reached ~ 0.5. Subsequently, the grown cells were pelleted and resuspended in infiltration buffer (10 mM MgCl_2_, 10 mM MES, and 150 µM acetosyringone, pH 5.6) to OD_600_ = 1.0 and incubated at room temperature (RT) for 3 h in the dark. The suspensions were infiltrated into the abaxial side of the *Nicotiana benthamiana* leaves. Then, plants were kept in the dark for 24 h at RT and then grown in a growth chamber for 48–72 h. GFP fluorescence was observed after 4 d of incubation using a BX53 microscope with a U-FBNA filter set (Evident, Tokyo, Japan).

### Transient dual-luciferase reporter assay in tobacco

A dual-luciferase reporter assay was performed in *Nicotiana benthamiana* leaves to assess the transcriptional activity of SlGLK2-CR4. The *SlLHCB2* promoter region reported as a direct target of SlGLK2 by Niu et al. (2022) was amplified using the primers listed in Table S1 and inserted into pGreenII 0800 (Hellens et al. 2000) linearized between *Xho*I and *Nco*I sites to generate the reporter construct. The pGII62-SK:sfGFP and pGII62SK-SlGLK2-CR4:sfGFP constructs, used in the subcellular localization experiment, served as the negative control and SlGLK2-CR4 effector constructs, respectively. Each construct was introduced separately into *Agrobacterium tumefaciens* GV3101 carrying pSoup-P19_K.

Agroinfiltration was performed as described above for the subcellular localization assay. The bacterial suspensions carrying the reporter and effector constructs were each adjusted to an OD_600_ = 0.5 and mixed at a reporter-to-effector volume ratio of 1:5 before infiltration. At 72 h after infiltration, approximately 5 mg of leaf tissue was collected from each of four independently infiltrated plants. The leaf tissue was homogenized in the cell lysis buffer supplied with the kit, and the resulting lysate was diluted tenfold before measurement. Firefly luciferase (LUC) and renilla luciferase (REN) activities were measured using a Dual Luciferase Reporter Assay Kit (Vazyme, Nanjing, China) and a Varioskan LUX multimode microplate reader (Thermo Fisher Scientific, Waltham, MA, USA), according to the manufacturer’s instructions. Relative reporter activity was calculated as the LUC/REN ratio and normalized to the mean value of the sfGFP control.

### Fruit morphological traits and biochemical analyses

Immature green fruit at 15 days after anthesis were harvested for total chlorophyll measurements, which were conducted according to a previous protocol (Ezura et al. 2024).

To standardize fruit load, the number of fruits was limited to two or three per truss and fewer than ten per plant, and fully ripe fruits at 10 days after breaker stage were harvested for morphological and quality measurements. Fruit weight was measured using an electronic balance, and fruit width and height were measured at the maximum equatorial diameter and along the longitudinal axis, respectively, using a digital caliper.

Lycopene and β-carotene contents were spectrophotometrically estimated essentially according to Nagata and Yamashita (1992), with minor modifications for small-scale extraction, microplate-based absorbance measurement, and optical path length correction. Carotenoids were extracted from 100 mg of homogenized fresh tomato fruit with 1.0 mL of acetone:hexane (4:6, v/v). After homogenization and centrifugation, the clear supernatant was diluted threefold with the same solvent mixture. Aliquots of 300 µL were transferred to an organic-solvent-resistant glass 96-well microplate (BC-MGPL96; Bio Medical Science Inc., Tokyo, Japan), and absorbance was measured at 453, 505, 645, 663, and 750 nm using a Varioskan LUX multimode microplate reader. Absorbance values were corrected for nonspecific absorbance at 750 nm and normalized to a 1-cm optical path length based on the theoretical path length of the sample in each well. Lycopene and β-carotene contents were calculated using the equations reported by Nagata and Yamashita (1992), corrected for dilution and sample fresh weight, and expressed as μg g⁻^1^ fresh weight.

Tocopherol contents were quantified essentially as described by Park et al. (2023), using 25 mg of freeze-dried tomato fruit powder by HPLC using a Prominence system (Shimadzu Corporation, Kyoto, Japan). The contents of α-, β-, γ-, and δ-tocopherol were converted to a fresh weight basis using the fresh-to-dry weight ratio, and expressed as μg g⁻^1^ fresh weight.

Total soluble solids (TSS) content was measured in ripe fruits with a refractometer Brix Meter BX-1 (Kyoto Electronics Manufacturing Co., Ltd., Japan).

### Transcriptomic analysis

The fruit of the immature green stage at 15 days after anthesis was horizontally dissected into three parts: stem, middle, and tip ends. Then, pericarps from the stem end of WT and *CR-Slglk2-4* fruit and from the tip end of *CR-Slglk2-4* fruit were harvested and immediately frozen in liquid nitrogen. Total RNA was extracted using a Plant RNeasy Extraction Kit (Qiagen, Germany), according to the manufacturer’s instructions. Each triplicate sample contained pericarps from at least three independent fruit. To remove residual genomic DNA, RNA samples were further purified using the RNA Clean & Concentrator Kit-25 (Zymo Research, Irvine, CA, USA) with DNase I treatment. RNA quality was checked using an Agilent 2100 Bioanalyzer (Agilent Technologies, Santa Clara, CA, USA). For RNA-seq analysis, mRNA was captured using the NEBNext Poly(A) mRNA Magnetic Isolation Module (New England Biolabs, Ipswich, MA, USA), and sequencing libraries were prepared using the NEBNext Ultra II Directional RNA Library Prep Kit (New England Biolabs, Ipswich, MA, USA) according to the manufacturer’s instructions. Paired-end sequencing (150 base pairs) for three replicates was performed using Illumina NovaSeq X Plus (Illumina, San Diego, CA, USA). Sequencing data were first cleaned using fastp ver. 0. 20.0 (Chen et al. 2018). Cleaned reads were pseudo-aligned to transcripts from the Tomato ITAG4.0 genome using Salmon (v1.9.0) (Patro et al. 2017). After processing using tximport (Soneson et al. 2015), differentially expressed genes (DEGs) between WT and mutants (*CR-Slglk2-4*) were identified using edgeR (Robinson et al. 2010) under iDEP 2.01 (Ge et al. 2018), applying a false discovery rate (FDR) cutoff of < 0.05. Gene Ontology (GO) term enrichment analysis was performed using ShinyGO v0.80 (Ge et al. 2020) with a default setting (FDR cutoff < 0.05) for significance, relative to the whole-transcriptome background. For the comparison with the DEGs in the *SlGLK2*-overexpression line (Nguyen et al. 2014), DEGs in the *SlGLK2*-overexpression line were re-analyzed using publicly available data (SRA079879) through the same workflow.

### Quantitative real-time PCR

Total RNA was extracted from stem end and tip end of immature green fruits at 15 days after pollination of unedited Micro-Tom (*u* allele) and *CR-Slglk2-4* line using an RNeasy plant mini kit (Qiagen, Venlo, The Netherlands). Each biological replicate consisted of at least three fruits, and three biological replicates were analyzed. After DNase I treatment, genomic DNA contamination was checked by PCR. Reverse transcription was conducted using ReverTra Ace qPCR RT Master Mix (Toyobo, Osaka, Japan). Quantitative real-time PCR (qRT-PCR) was conducted on a StepOne Real-Time PCR System (Applied Biosystems, CA, USA) using THUNDERBIRD NEXT qPCR Mix (Toyobo, Osaka, Japan) according to the manufacturer’s instructions. *SlCAC* (Solyc08g006960) was used as an internal control reference (Expósito-Rodríguez et al*.* 2008). Primer sequences used in this experiment are listed in Table S1. Relative expression levels were calculated using the 2^–ΔΔCT^ method (Livak and Schmittgen 2001), with the unedited Micro-Tom (*u* allele) stem end sample set to 1.

### Gene accession numbers

Accession numbers for the genes used in this study are: SlGLK2 (Solyc10g008160), *SlPORB* (Solyc10g006900), *SlMPEC* (Solyc10g077040), *SlGGDR* (Solyc03g115980), *SlCAB1C* (Solyc02g071010), *SlLHCB2* (Solyc07g047850), *SlLCYE* (Solyc12g008980), and CONSTANS-like (Solyc05g009310).

## Results

### Genome editing of uniform ripening (u) allele by temperature-tolerant *Lb*Cas12a

**Fig. 1:**
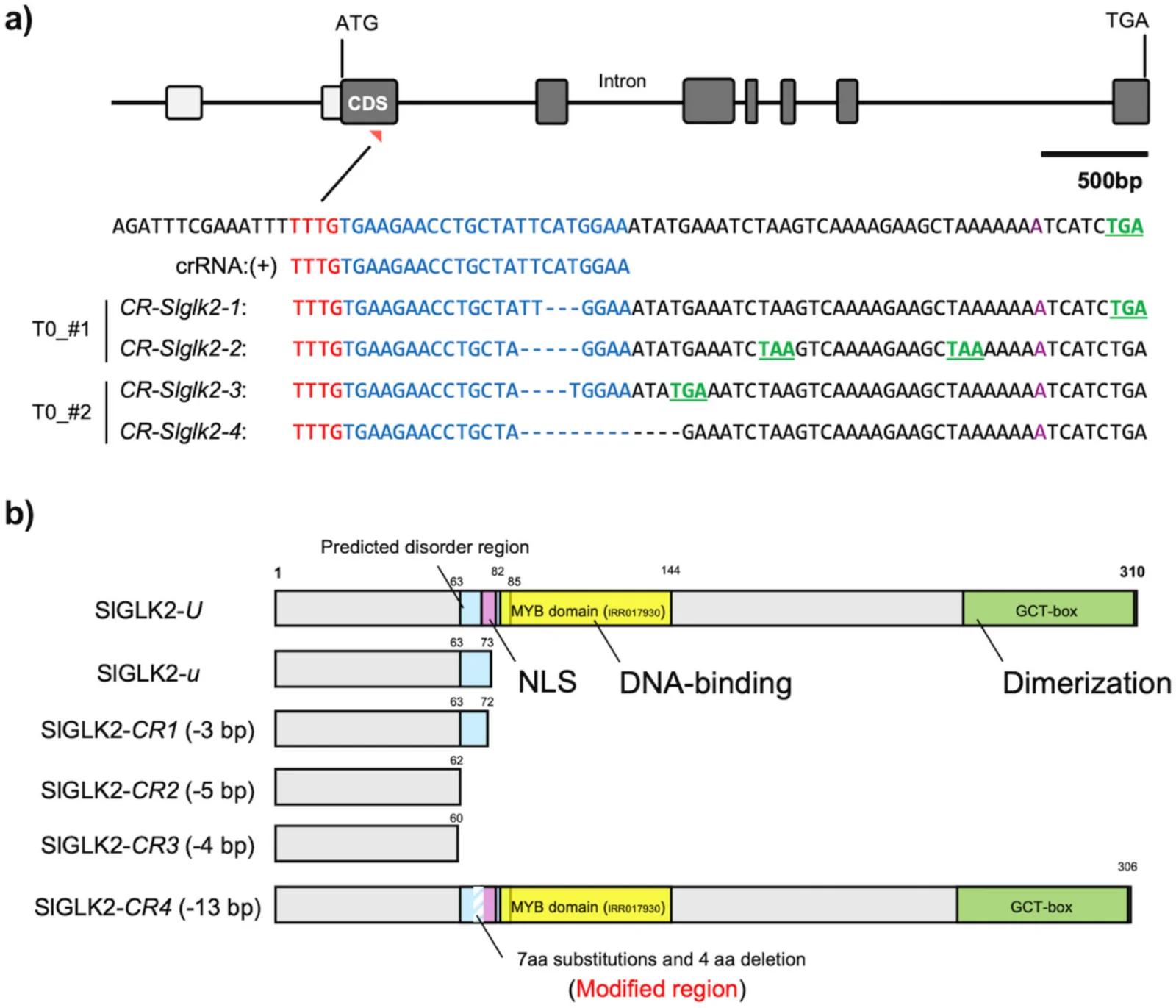
Additional mutation generated by tt*Lb*Cas12a system restores the reading frame of the tomato *uniform ripening* allele. **a** Structure of the *SlGLK2* gene and tt*Lb*Cas12a-generated mutations. The crRNA target sequence and protospacer-adjacent motif (PAM) sequences are highlighted in blue and red, respectively. Dashes indicate deleted nucleotides. Green bold, underlined letters indicate predicted in-frame stop codons, and purple letters indicate the single-base insertion present in the *uniform ripening* (*u*) allele.** b** Predicted protein domains of the functional *U* allele and mutant alleles with predicted disorder region (light blue), nuclear localization signal (pink), a DNA-binding MYB domain (IPR017930) (yellow), and dimerization domain GCT-box (green). aa, amino acids

To determine whether genome editing could restore the function of a gene lost during tomato improvement, the function of the *SlGLK2* gene was restored by re-editing the uniform ripening (u) allele, which is widely used in cultivated tomato (Powell et al. 2012). The *u* allele contains a single-base insertion in the coding region of *SlGLK2* (Fig. 1a). The single-base insertion causes a frameshift and premature truncation of SlGLK2, resulting in the loss of its DNA-binding and dimerization domains (Fig. 1b). AlphaFold3 predictions of full-length SlGLK2 support that the insertion site is located within a disordered region (Fig. S2). This prompted us to create a compensatory frameshift near the insertion site and restore the full-length reading frame. The insertion site was located in an A/T-rich sequence, which was difficult to target with the Cas9 protein (even with the NG-Cas9 type Cas9, data not shown). Therefore, we employed the Cas12a system, which is suitable for targeting A/T-rich regions (Li et al. 2020). In addition, a mutated version of a *Lachnospiraceae bacterium* Cas12a, called temperature-tolerant *Lb*Cas12a (tt*Lb*Cas12a), which has enhanced activity at low temperatures (Schindele and Puchta 2020) was used.

Genome editing of the tomato model cultivar Micro-Tom, which carries the non-functional *u* allele of SlGLK2, using tt*Lb*Cas12a generated three T_0_ lines carrying mutations near the target site. One of the three lines, *CR-Slglk2* #2, displayed green shoulders at the stem end of the mature green (MG) fruit, resembling the fruit phenotype of the functional *U* allele (Powell et al. 2012; Nguyen et al. 2014; Lupi et al. 2019). Direct sequencing of the T_0_ line #2 confirmed biallelic mutations, a 4-bp deletion (*CR-Slglk2-3* allele), and a 13-bp deletion (*CR-Slglk2-4* allele) (Fig. 1a). The *CR-Slglk2-4* allele caused a frameshift that resulted in a normal reading frame, whereas the *CR-Slglk2-3* allele caused a frameshift that resulted in another stop codon (Fig. 1b). Other alleles lacking reading frame restoration, a 3-bp deletion (*CR-Slglk2-1* allele) or a 5-bp deletion (*CR-Slglk2-2* allele), also produced fruit comparable to those of unedited Micro-Tom carrying the *u* allele plant.

**Fig. 2:**
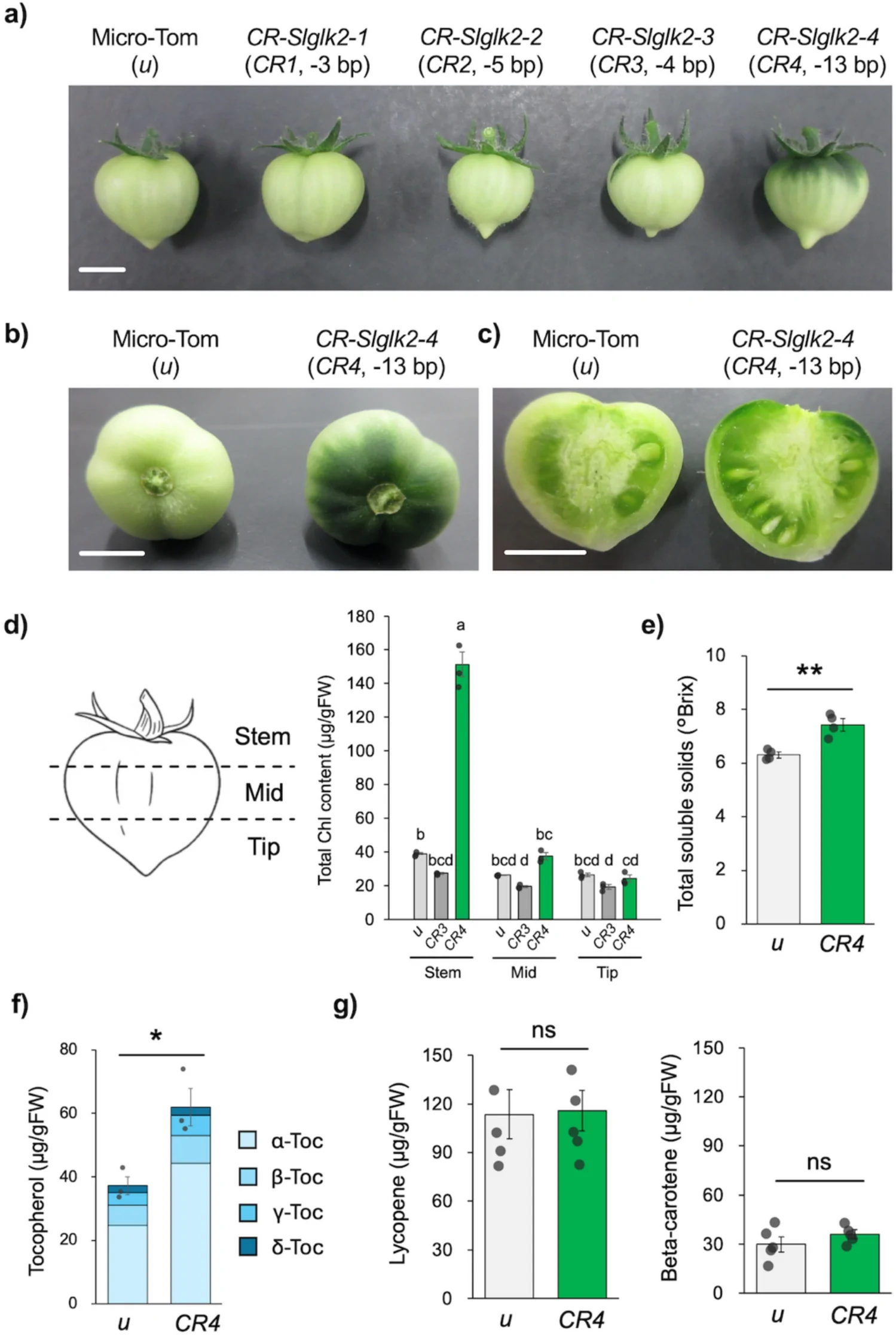
Genome editing of the u allele restores the green shoulder phenotype and enhances chlorophyll accumulation in tomato fruit. **a** Mature green (MG) fruits of unedited Micro-Tom (*u* allele) and four genome editing *Slglk2* mutant lines at 25 days after anthesis. Scale bar = 1 cm. **b** Stem end views of MG fruits of unedited Micro-Tom (*u* allele) and *CR-Slglk2-4* line (*CR4*, −13 bp) at 25 days after anthesis. Scale bar = 1 cm. **c** Longitudinal section of MG fruits of unedited Micro-Tom (*u* allele) and *CR-Slglk2-4* line at 25 days after anthesis. Scale bar = 1 cm. **d** Total chlorophyll content in the stem, middle, and tip regions of immature green (IMG) fruit at 15 days after anthesis. **e** Total soluble solids (TSS), **f** total tocopherol content, comprising α-, β-, γ-, and δ-tocopherols, and **g** major carotenoid contents (lycopene and β-carotene) of fully ripe fruit at 10 days after the breaker stage. Bars represent means ± SE, and dots indicate individual biological replicates. Sample sizes were *n* = 3 for panel (d), *n* = 4 for panel (e), *n* = 3 for panel (f), and *n* = 5 for panel (g). Different lowercase letters indicate significant differences among genotype–fruit region combinations (two-way ANOVA followed by Tukey’s HSD test, *p* < 0.05). Groups sharing at least one letter are not significantly different. For panels (e–g), asterisks indicate significant differences between the genotypes (an unpaired two-tailed Student’s *t*-test; * *p* < 0.05, ** *p* < 0.01; ns, not significant)

To confirm that the *CR-Slglk2-4* allele was responsible for the green shoulder phenotype, we obtained homozygous T_1_ lines and compared unedited Micro-Tom (*u* allele), *CR-Slglk2-3* line (*CR3*), and *CR-Slglk2-4* (*CR4*) plants. Fruits of the homozygous *CR-Slglk2-3* line, which carried a 4-bp deletion that did not restore the reading frame, showed a phenotype comparable to that of unedited Micro-Tom. In contrast, homozygous *CR-Slglk2-4* fruits, which carried the frame-restoring 13-bp deletion, produced green shoulder fruit similar to that observed in a T_0_ plant (Fig. 2a–c). *CR-Slglk2-4* fruits also accumulated significantly more chlorophyll at the immature green stage than unedited Micro-Tom and *CR-Slglk2-3* fruits (Fig. 2d). Fruit weight, width, and height were comparable among unedited Micro-Tom, *CR-Slglk2-3*, and *CR-Slglk2-4* plants (Fig. S3).

We next assessed quality-related traits in fully ripe unedited Micro-Tom and *CR-Slglk2-4* fruits. *CR-Slglk2-4* fruits showed significantly higher total soluble solids content than unedited Micro-Tom fruits (7.4 versus 6.3 °Brix; *p* = 0.0027; Fig. 2e). Total tocopherol content also increased significantly in *CR-Slglk2-4* fruits, from 37.25 to 62.05 μg g⁻^1^ fresh weight (*p* = 0.019; Fig. 2f). This increase mainly reflected higher α- and γ-tocopherol contents, whereas β- and δ-tocopherol contents did not differ significantly between the genotypes. In contrast, spectrophotometrically estimated lycopene and β-carotene contents did not differ significantly between unedited Micro-Tom and *CR-Slglk2-4* fruits (Fig. 2g).

Lastly, the mismatch-only off-target search detected only the intended target site. Under the bulge-permissive condition, we identified one additional candidate site containing three mismatches and a one-nucleotide RNA bulge, located 762 bp upstream of Solyc05g051420 (Fig. S4a). However, direct sequencing confirmed that the candidate off-target site remained unedited (Fig. S4b).

Together, these phenotypic and biochemical changes support functional recovery of SlGLK2 in *CR-Slglk2-4* following the introduction of the 13-bp deletion.

### Restoration of SlGLK2 function in *CR-Slglk2-4*

**Fig. 3:**
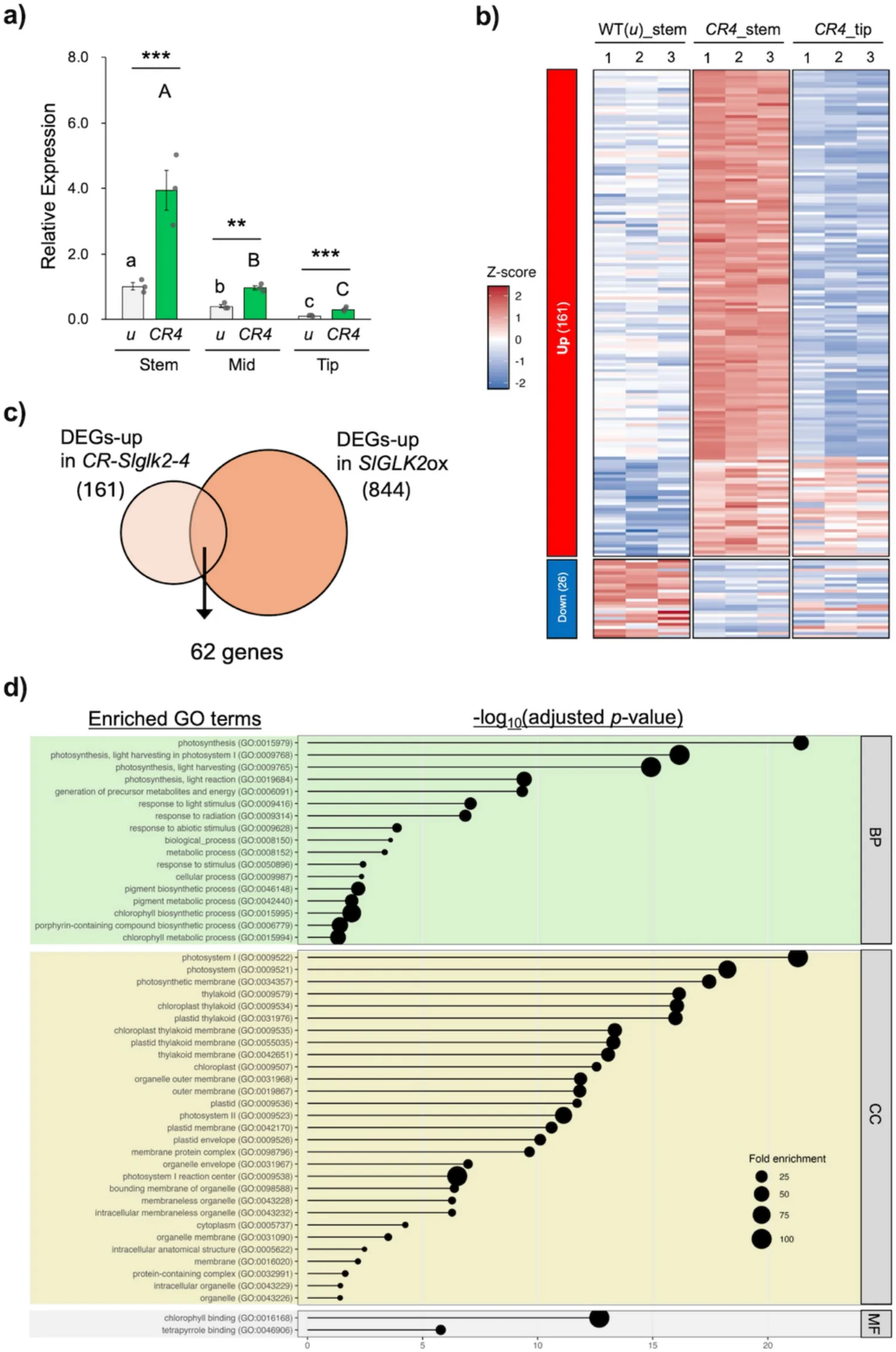
*SlGLK2*-associated downstream pathways are enhanced in the *CR-Slglk2-4* line. **a** Relative expression of *SlGLK2* in the stem, middle, and tip regions of unedited Micro-Tom (*u* allele) and *CR-Slglk2-4* (*CR4*) fruit at 15 days after anthesis. Bars represent means ± SE, and dots indicate individual biological replicates (*n* = 3). Asterisks indicate significant differences between the genotypes within each fruit region (unpaired two-tailed Student’s *t*-test; ** *p* < 0.01, *** *p* < 0.001; ns, not significant). Different lowercase letters denote significant differences among fruit regions within the *u* line, while different uppercase letters denote significant differences among fruit regions within *CR4* (one-way ANOVA followed by Tukey’s HSD, *p* < 0.05). **b** Heat map of differentially expressed genes (DEGs) in the stem end of *CR4* fruits.** c** Venn diagram showing commonly upregulated genes in *CR4* and *SlGLK2* overexpression (*SlGLK2ox*) fruits compared with the *u* line. **d** Gene Ontology (GO) overrepresentation analysis of the commonly upregulated genes in *CR4* and *SlGLK2*ox lines. Enriched GO terms in the Biological Process (BP), Cellular Component (CC), and Molecular Function (MF) categories are shown. The x-axis indicates −log10(adjusted *p* value), and point size represents fold enrichment of each GO term. Adjusted *p* values were calculated using Bonferroni correction for multiple testing, and terms with an adjusted* p* value < 0.05 were considered significant

*SlGLK2* expression exhibits a latitudinal gradient from the stem to the stylar end of the immature green fruit in tomato (Nguyen et al. 2014). This spatial gradient was maintained in *CR-Slglk2-4* fruits, with higher transcript levels at the stem end than at the stylar end (Fig. 3a). In addition, *SlGLK2* transcript levels were consistently higher in *CR-Slglk2-4* fruit than in unedited Micro-Tom at all examined positions, suggesting either an autoregulatory mechanism or a retrograde signal triggered by enhanced plastid development in the fruit. These results indicate that frame restoration recovered SlGLK2 protein function while preserving its characteristic spatial expression pattern. They also support the conclusion that the *uniform ripening* phenotype of the *u* allele primarily results from loss of SlGLK2 protein activity rather than an altered expression pattern.

To further validate the restoration of SlGLK2 function, we determined that the mutation did not affect the splicing patterns. Full-length cDNA sequences were amplified from fruit-derived mRNA of unedited Micro-Tom and *CR-Slglk2-4*, and their sequences were subsequently confirmed. According to the sequences and predicted protein sequences (Fig. S5), other than the mutation site, the results indicated that the *CR-Slglk2-4* mutation had no impact on the overall splicing patterns.

GLK transcription factors are GARP transcription activators with a MYB-like DNA-binding domain, a conserved GOLDEN2 C-terminal (GCT) box, and a nuclear localization signal (NLS) sequence in the upstream region of the MYB-like DNA-binding domain (Hall et al. 1998; Rossini et al. 2001; Fitter et al. 2002). The comparison of amino acid sequences and predicted structures showed that the *CR-Slglk2-4* mutation was located upstream of the MYB-like DNA-binding domain, in a region poorly conserved among plant orthologs (Fig. S5). NLS predictions using LOCALIZER (Sperschneider et al. 2017) and NLStradamus (Nguyen Ba et al. 2009) confirmed that the additional mutation did not alter the NLS signal upstream of the MYB-like domain (Fig. S7). Here, SlGLK2-*U*, SlGLK2-*u*, and SlGLK2-*CR4* denote the proteins encoded by the functional *U* allele, non-functional *u* allele, and *CR-Slglk2-4* allele, respectively. Consistently, the sfGFP fusion proteins of SlGLK2-*U* and SlGLK2-*CR4* were localized to the nucleus, whereas no signal of SlGLK2-*u* was detected (Fig. S8). Collectively, these results indicate that the *CR-Slglk2-4* allele restored nuclear localization of its translated protein.

### Restoration of SlGLK2-associated gene expression patterns in *CR-Slglk2-4* fruits

To determine whether SlGLK2-*CR4* variant in the *CR-Slglk2-4* line regulates a gene set similar to functional SlGLK2, transcriptome analysis was conducted using stem or tip end tissue from immature green fruits of *u* allele and *CR-Slglk2-4* plants (Table S2–S4). In comparison with the *u* allele, 161 genes were upregulated, and 26 genes were downregulated in the stem end of *CR-Slglk2-4* fruit (Fig. 3b). Of the 161 upregulated genes, 62 (38.5%) overlapped with differentially upregulated genes in the *SlGLK2*-overexpression line (Fig. 3c, Table S5), based on reanalysis of published RNA-seq data (Nguyen et al. 2014), whereas only three genes were commonly downregulated (Table S6). Notably, 129 of the 161 genes (80.1%) exhibited higher expression in the stem end than tip end of *CR-Slglk2-4* fruit, suggesting their gradient expression pattern. Furthermore, 34 out of the 62 genes (54.8%) were identified as potential direct targets of SlGLK2 (Table S6), exhibiting significant binding signals in prior chromatin immunoprecipitation sequencing (ChIP-seq) analysis (Tu et al. 2022).

**Fig. 4:**
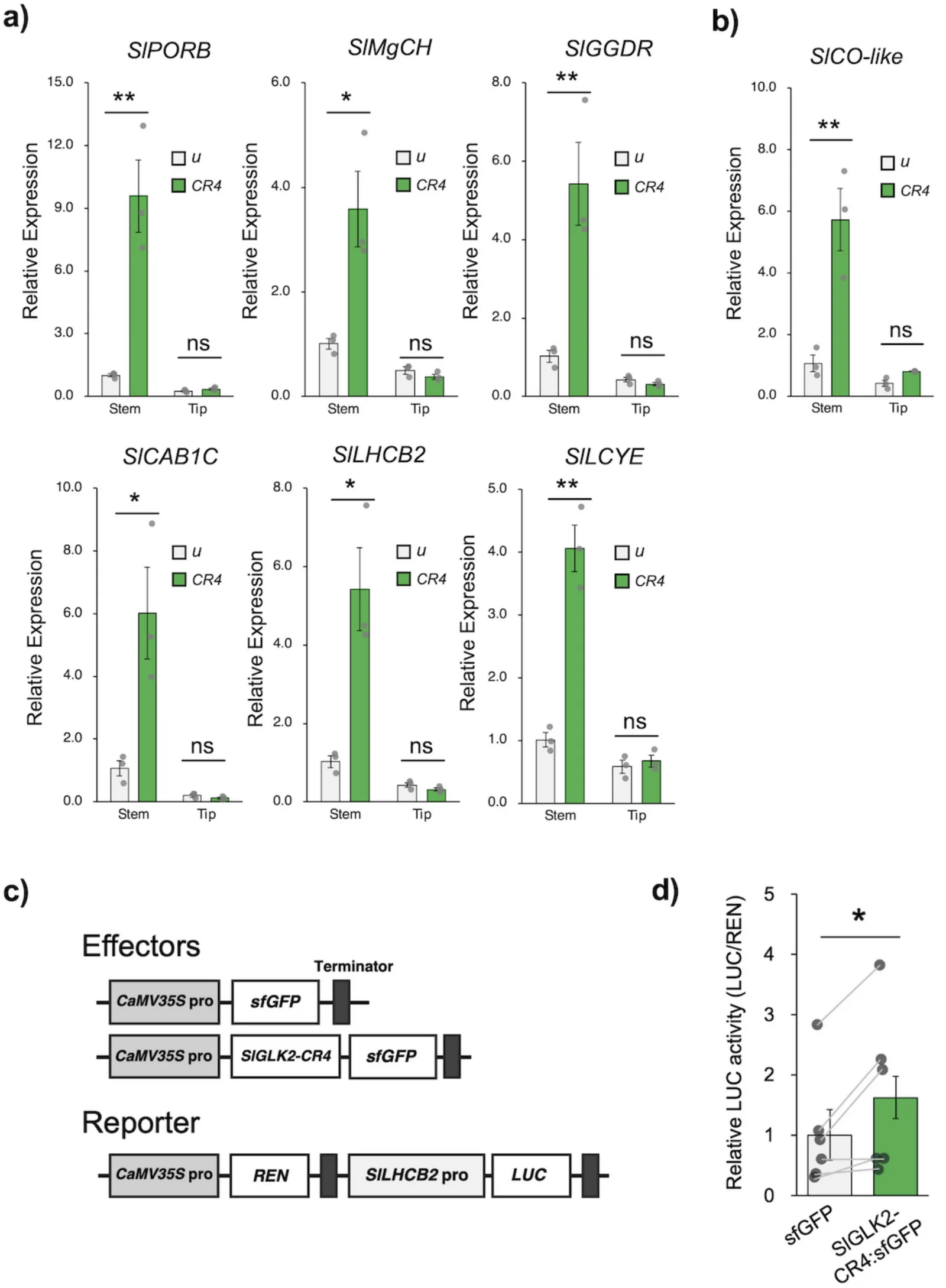
Validation of SlGLK2 target gene expression and transcriptional activity in *CR-Slglk2-4* fruits. Validation of expression of common DEGs by quantitative RT-PCR (qRT-PCR). qRT-PCR analysis validates the expression patterns of putative SlGLK2-targeted genes involved in chlorophyll biosynthesis and plastid biogenesis **(a)** and a transcription factor **(b).** Bars represent means ± SE, and dots indicate individual biological replicates (*n* = 3). Asterisks indicate significant differences between the genotypes within each fruit region (an unpaired two-tailed Student’s *t*-test; ** *p* < 0.01, *** *p* < 0.001; ns, not significant). *SlPORB*, protochlorophyllide reductase; *SlMPEC*, *Mg-protoporphyrin IX monomethyl ester cyclase*; *SlGGDR*, geranylgeranyl diphosphate reductase; *SlCAB1C*, chlorophyll a-b binding protein 1C; *SlLHCB2*, light-harvesting complex B2; *SlLCYE*, lycopene ε-cyclase; *SlCO-like*, CONSTANS-like. **c** Schematic illustration of the effector and reporter constructs. The *SlLHCB2* promoter fragment was inserted into the pGreenII 0800-LUC vector to generate the reporter construct. The effector construct contained SlGLK2-CR4 fused to sfGFP under the control of the *CaMV 35S* promoter. The sfGFP construct served as the negative control. *CaMV35S* pro, cauliflower mosaic virus 35S promoter; *SlLHCB2* pro, *SlLHCB2* promoter; *LUC*, firefly luciferase gene; *REN*, Renilla luciferase gene; sfGFP, superfolder green fluorescent protein. **d** Relative reporter activity. Reporter activity (LUC/REN) normalized to the mean value of the sfGFP control. Paired values obtained from the same leaf are connected by lines. Bars represent means ± SE, and dots indicate individual paired leaves (*n* = 6). Asterisk indicates a significant difference (a two-tailed Wilcoxon signed-rank test, *p* < 0.05)

Gene ontology analysis of the 62 commonly upregulated gene set showed overrepresentation of biological process and cellular component terms involved in chloroplast- and photosynthesis-associated processes, including “chlorophyll biosynthetic process (GO:0015995)”, “photosynthesis, light harvesting in photosystem I (GO:0009768)”, “photosystem I reaction center (GO:0009538)”, and “chloroplast thylakoid lumen (GO:0009543)” (Fig. 3d; Table S7). To further validate these transcriptional changes, we tested the expression of the commonly upregulated genes with SlGLK2-binding signal in the previous ChIP-seq analysis (Tu et al. 2022). Seven genes present in both datasets were selected for quantitative RT-PCR analysis, which confirmed their elevated expression in *CR-Slglk2-4* fruits (Fig. 4a). These genes included key components of chlorophyll biosynthesis and light-harvesting complexes, such as protochlorophyllide reductase B (*SlPORB*), Mg-protoporphyrin IX monomethyl ester cyclase (*SlMPEC*), geranylgeranyl diphosphate reductase (*SlGGDR*), chlorophyll a-b binding protein 1C (*SlCAB1C*), light-harvesting complex B2 (*SlLHCB2*). In addition, *lycopene epsilon-cyclase* (*SlLCYE*), recently proposed as a potential SlGLK2-regulated gene in the carotenoid pathway (Sun et al. 2025), was also upregulated in *CR-Slglk2-4*. Notably, one transcription factor, CONSTANS-like zinc finger protein (Solyc05g009310), also displayed elevated expression (Fig. 4b).

Lastly, to determine whether the frame-restored SlGLK2-CR4 protein retained transcriptional activation activity, we performed a transient dual-luciferase reporter assay in *Nicotiana benthamiana* leaves using the promoter of *SlLHCB2* (Solyc07g047850), a previously reported direct target of SlGLK2. Co-expression of SlGLK2-CR4 significantly increased the LUC/REN ratio compared with the sfGFP control, indicating that SlGLK2-CR4 can activate the *SlLHCB2* promoter (Fig. 4c, d). These results support the recovery of transcriptional activation activity in the frame-restored SlGLK2-CR4 protein.

## Discussion

**Fig. 5:**
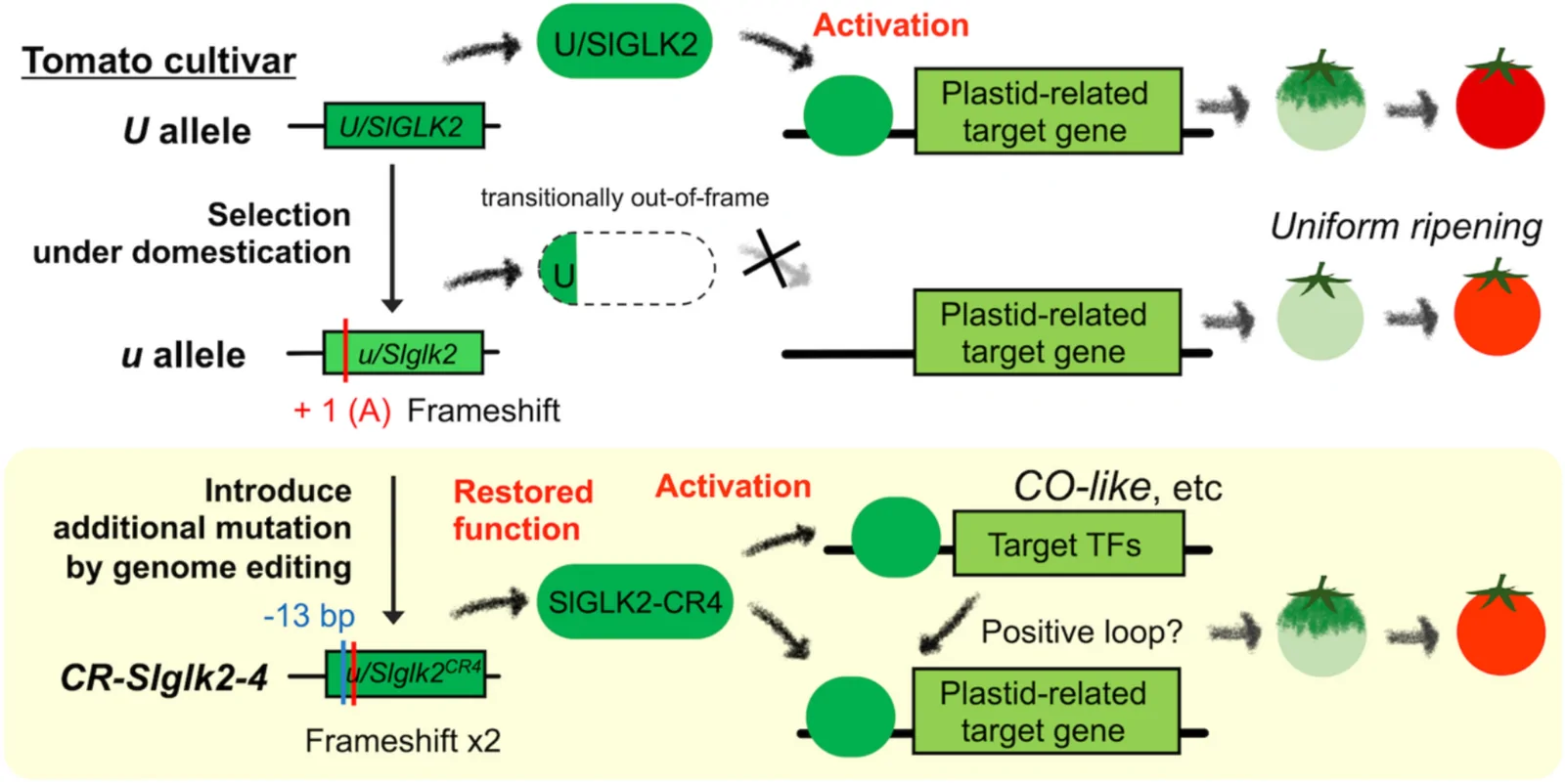
Schematic summary illustrating restoration of SlGLK2 function by genome editing of the *u* allele. The processes of plant domestication and crop improvement have inadvertently resulted in trade-offs that affect crop quality. In cultivated tomato (*Solanum lycopersicum*) varieties, the *uniform ripening* (*u*) allele, which contains a single-base insertion that disrupts the reading frame of *SlGLK2*, has been extensively selected to enhance fruit appearance. However, this mutation reduced the accumulation of sugars, tocopherols, and carotenoids due to impaired plastid development. This study employed a simple CRISPR/Cas system to introduce an additional mutation into the non-functional allele, thereby restoring the function of the lost gene by correcting its reading frame. The 13-bp deletion in the *u* allele reinstated *SlGLK2* function without altering its spatial expression in fruit and activated the downstream pathway, resulting in recovery of the green shoulder phenotype and increases in total soluble solids and tocopherol contents

In this study, we used an SDN1-category genome editing approach to recover the function of an improvement-associated non-functional *SlGLK2* allele (Fig. 5). The introduction of a compensatory frameshift into the *u* allele generated an in-frame SlGLK2 variant that showed transcriptional activity, reactivated the downstream transcriptional program associated with plastid biogenesis, and recovered the green shoulder fruit phenotype. In addition, the edited line showed higher total soluble solids and total tocopherol content in ripe fruit, whereas lycopene and β-carotene contents, fruit weight, and fruit size were unchanged. These results indicate that restoration of SlGLK2 function selectively enhanced several fruit-quality-related traits without substantially altering fruit morphology. These results demonstrate the proof of concept that conventional SDN1-based genome editing can be used not only for gene disruption but also to reactivate dormant genes that were lost during plant domestication or crop improvement.

### The re-editing of improvement-derived, non-functional alleles as a strategy to reactivate dormant genes

Domesticated and improved plants unintentionally lose beneficial genes during plant domestication or improvement processes. Non-processed pseudogenes arise from duplication in genomic DNA and subsequent disablement, most commonly through disruptive frameshift mutations or premature stop codon formation (Akhunov et al. 2013). Although many pseudogenes have lost their function at the translational level due to a frameshift-associated premature stop codon and reduced or undetectable expression levels of their transcripts, a considerable number of pseudogenes are also transcribed (Pink et al. 2011; Sisu et al. 2020; Feng et al. 2022). In tomato, the protein function of the *SlGLK2* gene was lost due to truncation of the translated protein by a premature stop codon, whereas the *SlGLK2* transcripts are still detectable (Powell et al. 2012). Early tomato domestication and improvement have been focused on fruit appearance and yield rather than stress tolerance and fruit nutrition (Powell et al. 2012; Bolger et al. 2014; Tieman et al. 2017; Li et al. 2024). For instance, widely cultivated varieties produce large fruits but are substantially more sensitive to salt stress than their wild ancestors. A major variant, an in-frame, six-base insertion in the coding sequence of the *SlHAK20* gene, which encodes a clade IV HAK/KUP/KT transporter, is associated with Na^+^/K^+^ homeostasis and mediates salt tolerance in tomato (Wang et al. 2020). The C-to-T substitution at nucleotide position 265 of *SlBBX18* was selected during tomato evolution, increasing drought sensitivity due to premature stop codon formation (Li et al. 2024). Furthermore, a deleterious mutation in the *SUPPRESSOR OF SP2* (*SSP2*) gene in cultivated tomato was repaired by base editing, resulting in early fruit yield (Glaus et al. 2025). To our knowledge, this study provides the first proof of concept that a simple SDN1-induced compensatory frameshift can generate a functional in-frame allele from a domestication- or improvement-associated loss-of-function allele. In addition to the introduction of gain-of-function mutations targeting transcriptional regulatory regions (Rodríguez-Leal et al. 2017; Oliva et al. 2019; Wang et al. 2021), this approach may provide an alternative method for recovering useful allelic functions or enhancing target traits directly in cultivated genetic backgrounds.

Conventional introgression from wild or ancestral accessions can introduce undesirable linked regions through linkage drag. By contrast, the strategy presented here modifies the pre-existing allele directly within the cultivated genetic background and therefore provides a potential route to recover useful gene functions without large-scale introgression. Because this approach requires neither a repair template nor precise restoration of the wild-type sequence, it may offer a technically accessible option when limited amino acid changes are tolerated. Its broader applicability will depend on the location and type of the original mutation, available target sites, repair outcomes, and the structural tolerance of the encoded protein. Testing additional genes, mutation types, and commercial cultivars will help define the range of applications for this reframing strategy.

### Putative downstream factors associated with SlGLK2-dependent transcriptional programs in tomato fruit

The GLK family functions as a central regulator of plastid development and coordinates a wide range of plastid-associated processes (Fitter et al. 2002; Waters et al. 2009; Zubo et al. 2018; Hernández‐Verdeja and Lundgren 2024). In tomato, chloroplasts differentiate into chromoplasts, accumulating health-promoting nutrients such as carotenoids (Sadali et al. 2019; Gong et al. 2024). Enhancing plastid development in fruits is a promising approach for improving the nutritional quality of fruit-bearing crops (Wang et al. 2008; Alves et al. 2020). However, the underlying processes of plastid development and differentiation, particularly their regulatory mechanisms, in developing fruits remain unclear.

Our comparative transcriptomic analysis identified 62 genes that were commonly upregulated in both *CR-Slglk2-4* and *SlGLK2ox* fruits, representing potential downstream targets of the SlGLK2-regulated transcriptional pathway in developing fruit. Notably, 34 of these genes, including well-known photosynthesis-related genes and several regulatory factors, overlapped with genes identified in the previous ChIP-seq analysis (Tu et al. 2022), making them candidates for direct regulation by SlGLK2.

Consistent with the transcriptomic results, the frame-restored SlGLK2-CR4 protein activated the *SlLHCB2* promoter in the transient dual-luciferase assay. *SlLHCB2* is a previously identified direct target of SlGLK2 (Niu et al. 2022) and was also upregulated in *CR-Slglk2-4* fruit. These results provide functional evidence that the reframed SlGLK2 protein retains transcriptional activity toward a representative photosynthesis-related target gene. Although the activity of SlGLK2-CR4 was not directly compared with that of the protein encoded by the functional *U* allele, the reporter assay, together with the transcriptomic and phenotypic data, supports the functional recovery of SlGLK2 in *CR-Slglk2-4*.

Among the commonly upregulated genes, we identified a CONSTANS-like zinc finger protein (Solyc05g009310), which is homologous to Arabidopsis Class III BBX subfamily members (BBX14/15/16/17) (Khanna et al. 2009). In Arabidopsis, BBX16, which directly promotes the expression of SUPERROOT 2 (SUR2), which encodes a suppressor of auxin biosynthesis (﻿Zhang et al. 2014), is directly regulated by GLK1 during seedling photomorphogenesis (Veciana et al. 2022). Additionally, BBX14 negatively regulates nitrogen starvation- and dark-induced leaf senescence (Buelbuel et al. 2023). Consistent with enhanced maintenance of photosynthetic functions, several senescence-downregulated genes, including light-harvesting complex and *PORB*, showed high expression in *CR-Slglk2-4* fruit. In tomato, CONSTANS-like transcription factors, such as SlCOL1 (Solyc02g089540) and SlBBX24 (Solyc06g073180), have been shown to stabilize SlGLK2 protein, thereby enhancing chlorophyll biosynthesis in fruits (Cui et al. 2024). Considering that a limited number of transcriptional regulators were commonly induced in *CR-Slglk2-4* and *SlGLK2ox* fruits, Solyc05g009310 represents a candidate secondary regulator within the SlGLK2-associated transcriptional networks. Its role in plastid biogenesis, however, remains to be experimentally determined. Future studies on isolated candidate genes will further elucidate the regulatory mechanisms underlying plastid biogenesis during tomato fruit development.

In addition to its downstream targets, *SlGLK2* itself showed increased transcript levels in *CR-Slglk2-4* fruit. Since previous ChIP-seq analysis did not identify clear SlGLK2 binding at *SlGLK2* locus (Tu et al. 2022), this increase may reflect indirect regulation. Notably, the spatial pattern of *SlGLK2* expression paralleled chlorophyll accumulation along the stem-to-tip axis, suggesting that local developmental or plastid-associated signals may influence SlGLK2 expression in fruits.

### *CR-Slglk2-4* as a model resource for studying plastid biogenesis in tomato fruit

The miniature cultivar Micro-Tom has been widely used as a model system for studying fruit physiology and development due to its small plant size, shorter life cycle, and extensive mutant resources (Carvalho et al. 2011; Shikata and Ezura 2016; Gasparini et al. 2025). However, Micro-Tom carries the *u* allele (Carvalho et al. 2011), which limits its utility for study of chloroplast development in fruit. In the *CR-Slglk2-4* line, plastid development in fruit is restored within the genetic background of cultivated tomato, resulting in a pronounced green shoulder phenotype. Importantly, this restoration reflects functional recovery of SlGLK2 without introducing transgenes or large-scale genomic modifications. As *CR-Slglk2-4* retains the advantageous characteristics of Micro-Tom while exhibiting spatially distinct plastid development within a single fruit, this line provides a useful genetic resource for investigating the regulatory mechanisms underlying plastid biogenesis and its spatial modulation during tomato fruit development.

Collectively, our results show that an SDN1-induced secondary frameshift can generate a functional in-frame allele from a pre-existing loss-of-function allele. This reframing strategy expands the conventional use of nuclease-based genome editing from gene disruption to functional gene reactivation. Although its efficiency and applicability will depend on the original mutation and the structural tolerance of the target protein, the approach provides a simple and potentially useful option for recovering valuable allelic functions in crop genomes.

## Supplementary Information

Below is the link to the electronic supplementary material.

Supplementary file1 (DOCX 2234 KB)

Supplementary file2 (XLSX 4662 KB)

## Data Availability

Enquiries about data and materials availability should be directed to the corresponding author. The raw transcriptomic data generated in this study have been deposited in the NCBI Sequence Read Archive (SRA) under BioProject accession number PRJNA1513752.
