# Supplementary material for: Reactivation of a loss-of-function SlGLK2 allele by frame-restoring genome editing in tomato
Source: Plant Cell Rep. 2026 Oct 15;45(11):333. doi: 10.1007/s00299-026-04024-z (PMC13672529; doi:10.1007/s00299-026-04024-z)
Supplement: Supplementary file 1 [file 299_2026_4024_MOESM1_ESM.docx]

**Supporting information**

**Fig. S1** Schematic view of the genome editing construct used

ttLbCas12a, temperature-tolerant *Lachnospiraceae bacterium* Cas12a (*Lb*Cas12a). PcUbi, Parsley ubiquitin promoter; Tpea3A, terminator of ribulose-1,5-bisphosphate carboxylase small subunit; AtU6-26, *Arabidopsis thaliana* U6-26 promoter; crRNA, sequence of CRISPR RNA; PNOS, NOS promoter; NTPII, Neomycin phosphotransferase II gene; TNOS, NOS terminator; LB, left border; RB. right border.

**Fig. S2** Alignment of protein sequences of functional *U* allele, non-functional u allele, and four CRISPR-generated alleles

Listed sequences were predicted from the sequenced cDNA nucleotide sequences. Amino acid substitutions relative to the functional *U* allele are shown in red.

**Fig. S3** Fruit morphological traits of unedited Micro-Tom and homozygous *CR-Slglk2* lines

Fruit weight **(a)**, fruit width **(b)**, and fruit height **(c)** were measured in fully ripe fruits of unedited Micro-Tom (*u*), *CR-Slglk2-3* (*CR3*), and *CR-Slglk2-4* (*CR4*) plants. Bars represent means ± SE, and dots indicate individual fruits (n = 30). Statistical differences among genotypes were evaluated using one-way ANOVA followed by Tukey’s honestly significant difference (HSD) test. No significant differences were detected for any trait (*p* > 0.05).

**Fig. S4** Prediction and sequence validation of a potential off-target site for the tt*Lb*Cas12a crRNA targeting SlGLK2. **(a)** Alignment of the intended SlGLK2 target sequence and the predicted off-target candidate sequence located 762 bp upstream of Solyc05g051420. Mismatched nucleotides and the predicted one-nucleotide RNA bulge are indicated. **(b)** Sanger sequencing chromatograms of the predicted off-target candidate site in unedited Micro-Tom and the *CR-Slglk2-4* line. No sequence alteration was detected at this site.

**Fig. S5** Alignment of the amino acid sequences around the mutated region of SlGLK2 and homologs from other plant species

These sequences have diversified lengths and sequences in this region. Amino acid sequences of SlGLK2 homologs were aligned and displayed by NCBI Multiple Sequence Alignment Viewer (Version 1.22.0). Disordered region (light blue), target region (pink), MYB domain (yellow), GCT box (green). *Solanum lycopersicum* (NP_001266252.1), *Solanum pennellii* (XP_015089237.1), *Solanum tuberosum* (KAH0722065.1), *Capsicum annuum* (AFF60406.1), *Smallanthus sonchifolius* (KA3774048.1), *Gossypium mustelinum* (TYI95756.1), *Morella rubra* (KAB1227988.1), *Trifolium pratense* (XP_045804791.1),  *Nicotiana tomentosiformis* (XP_009589600.1), *Medicago truncatula* (XP_013465151.1), *Citrus sinensis* (XP_006470159.1), *Glycine max* (NP_001241943.1), *Glycine soja* (KAG4967779.1), *Pisum sativum* (XP_050883705.1)

**Fig. S6** The mutated region of *CR-Sglk2-4* allele is located in the linker region of SlGLK2

Predicted SlGLK2 structures of the functional *U* allele and *CR-Sglk2-4* allele (CR4) using AlphaFold3. The target region and modified sequences are highlighted in green and blue, respectively. Predicted MYB domain is shown in yellow.

**a)**

**b)**

**Fig. S8** *CR-Sglk2-4* allele does not affect nuclear localization signal

Subcellular localization prediction of SlGLK2 variants using LOCALIZER v.1.0.4 (https://localizer.csiro.au/) indicates that both variants, *U* allele (a) and 13-bp-deletion variant (b) of the *CR-Slglk2-4* (*CR4*) line, have a nuclear localization signal with no transit peptides.

**Fig. S8** Subcellular localization of SlGLK2 variants in *Nicotiana benthamiana* leaf

Subcellular localization of the functional *U* allele, non-functional *u* allele, and *CR-Slglk2-4* (CR4) variant were fused to superfolder green fluorescent protein (sfG) and transiently expressed in *Nicotiana benthamiana* leaves.
